# Upregulation of inducible NO synthase by exogenous adenosine in vascular smooth muscle cells activated by inflammatory stimuli in experimental diabetes

**DOI:** 10.1186/s12933-016-0349-x

**Published:** 2016-02-16

**Authors:** Alberto Nassi, Francesca Malorgio, Serena Tedesco, Andrea Cignarella, Rosa Maria Gaion

**Affiliations:** Department of Pharmaceutical and Pharmacological Sciences, University of Padova, Padova, Italy; Department of Medicine, University of Padova, Padova, Italy; Transplant Immunology Unit, Padua University Hospital, Padova, Italy

**Keywords:** Adenosine, iNOS, Smooth muscle cells, Vasorelaxation, Purine turnover

## Abstract

**Background:**

Adenosine has been shown to induce nitric oxide (NO) production via inducible NO synthase (iNOS) activation in vascular smooth muscle cells (VSMCs). Although this is interpreted as a beneficial vasodilating pathway in vaso-occlusive disorders, iNOS is also involved in diabetic vascular dysfunction. Because the turnover of and the potential to modulate iNOS by adenosine in experimental diabetes have not been explored, we hypothesized that both the adenosine system and control of iNOS function are impaired in VSMCs from streptozotocin-diabetic rats.

**Methods:**

Male Sprague–Dawley rats were injected with streptozotocin once to induce diabetes. Aortic VSMCs from diabetic and nondiabetic rats were isolated, cultured and exposed to lipopolysaccharide (LPS) plus a cytokine mix for 24 h in the presence or absence of (1) exogenous adenosine and related compounds, and/or (2) pharmacological agents affecting adenosine turnover. iNOS functional expression was determined by immunoblotting and NO metabolite assays. Concentrations of adenosine, related compounds and metabolites thereof were assayed by HPLC. Vasomotor responses to adenosine were determined in endothelium-deprived aortic rings.

**Results:**

Treatment with adenosine-degrading enzymes or receptor antagonists increased iNOS formation in activated VSMCs from nondiabetic and diabetic rats. Following treatment with the adenosine transport inhibitor NBTI, iNOS levels increased in nondiabetic but decreased in diabetic VSMCs. The amount of secreted NO metabolites was uncoupled from iNOS levels in diabetic VSMCs. Addition of high concentrations of adenosine and its precursors or analogues enhanced iNOS formation solely in diabetic VSMCs. Exogenous adenosine and AMP were completely removed from the culture medium and converted into metabolites. A tendency towards elevated inosine generation was observed in diabetic VSMCs, which were also less sensitive to CD73 inhibition, but inosine supplementation did not affect iNOS levels. Pharmacological inhibition of NOS abolished adenosine-induced vasorelaxation in aortic tissues from diabetic but not nondiabetic animals.

**Conclusions:**

Endogenous adenosine prevented cytokine- and LPS-induced iNOS activation in VSMCs. By contrast, supplementation with adenosine and its precursors or analogues enhanced iNOS levels in diabetic VSMCs. This effect was associated with alterations in exogenous adenosine turnover. Thus, overactivation of the adenosine system may foster iNOS-mediated diabetic vascular dysfunction.

## Background

The pathophysiology of vascular disease associated with diabetes mellitus involves abnormalities in the functions of resident and circulating cells [1] as well as low-grade vascular inflammation with increased cytokine release. Diabetes is associated with altered expression and function of adenosine receptors, which are expressed on most cell types in the cardiovascular system [2]. Increased activity of the adenosine-degrading enzyme adenosine deaminase (ADA) has been also reported in diabetic patients [3] and experimental animals [4], suggesting that alterations in the adenosine system contribute to the pathophysiology of the disease.

Vascular smooth muscle cells (VSMCs) have an active adenosine metabolism [5, 6], and express all known adenosine receptors [7]. Under conditions such as hypoxia, ischaemia or inflammation, the level of extracellular adenosine increases dramatically to improve blood flow via vasodilator A_2A_ and A_2B_ receptors expressed on the endothelium and smooth muscle [8]. Adenosine that accumulates in the extracellular compartment, thus reaching its cell surface receptors, can be released as such by means of bidirectional equilibrative nucleoside transporters (ENTs), or formed extracellularly from cell-derived nucleotides that are dephosphorylated by enzymes bound to the external surface of plasma membrane. Among these, ecto-5′ nucleotidase (CD73) promotes the final conversion of AMP into adenosine. On the other hand, metabolic conversion by ecto-ADA and transport into cells mediated by ENTs and/or concentrative nucleoside transporters account for adenosine removal from the extracellular space [9, 10]. In VSMCs, 95 % of adenosine transport is mediated by ENT-1 and the rest by ENT-2 [10].

Sustained production of nitric oxide (NO) through the inducible form of NO synthase (iNOS) is a response to a variety of agents including proinflammatory cytokines [11]. VSMCs are endowed with the metabolic machinery that allows release of large amounts of NO in response to cytokines and endotoxin [12–16]. The ability of adenosine to increase basal as well as cytokine- or lipopolysaccharide (LPS)-stimulated NO release in VSMCs is documented [17–19], and it is interpreted as a potential beneficial effect, inasmuch as it would prevent pathologic vaso-occlusion triggered by other local mediators [20, 21]. On the other hand, the augmented release of NO derived from iNOS is involved in the development of diabetic vascular complications [22–24].

We therefore hypothesized that experimental type I diabetes would alter the modulation of iNOS protein synthesis and activity by exogenous adenosine and agents affecting its turnover in VSMCs. We tested this hypothesis by isolating VSMCs from aortas of diabetic and nondiabetic rats, stimulating VSMCs with LPS combined with a cytokine cocktail in the presence or absence of adenosine and related compounds, and assessing iNOS functional expression as well as adenosine turnover. Our results indicated that, while endogenous adenosine attenuated iNOS production, supplementation with exogenous adenosine was associated with abnormal purine turnover and enhanced iNOS levels in diabetic VSMCs, which may foster diabetic vascular dysfunction.

## Methods

### Chemicals and antibodies

Streptozotocin (STZ) and MRS 1730 were purchased from Tocris Cookson. The anti-iNOS antibody was from BD Biosciences, whereas the peroxidase-coupled secondary antibody was obtained from Vector. Recombinant rat cytokines were obtained from Tebu-Bio. Unless otherwise specified, chemicals were purchased from Sigma-Aldrich.

### Animals

Male Sprague-Dawley rats weighing 200–250 g (Charles River, Italy) were kept in temperature-controlled facilities on a 12-hour light-dark cycle and fed normal chow. At the time of cell or tissue harvest, animals were sacrificed by asphyxia using carbon dioxide. The work described has been carried out in accordance with the EU Directive 2010/63/EU for animal experiments (http://ec.europa.eu/environment/chemicals/lab_animals/legislation_en.htm).

### Diabetes induction

Diabetes was induced by a single i.p. injection of 65 mg/Kg STZ freshly dissolved in 50 mM citrate buffer (pH 4.5). Blood glucose was monitored weekly after STZ injection using Glucurtest (Roche Diagnostics). Animals were diabetic for 3 weeks prior to aortic harvest and vascular cell isolation. Only hyperglycemic rats with fasting blood glucose ≥11.1 mM were selected for experiments.

### Cell culture

VSMCs from aortic intimal-medial layers were isolated and cultured as previously described [15, 25]. Briefly, VSMCs were synchronized in medium containing 0.4 % FCS for 24 h and incubated for a further 24 h where indicated with a cytokine mixture comprising 10 ng/mL interleukin (IL)-1β, 10 ng/mL interferon (IFN)-γ and 25 ng/mL tumor necrosis factor (TNF)-α plus 1 µg/mL LPS. Such a mixture consistently induces iNOS protein formation in VSMCs [25].

### Western blotting

Cultured VSMCs were washed twice with phosphate-buffered saline and extracted directly into lysis buffer as described elsewhere [26]. At least 30 µg cell protein were loaded onto 10 % SDS-acrylamide gels. Following electrophoresis, proteins were transferred onto Hybond-ECL membranes (GE Healthcare), which were incubated in blocking solution for 2 h and then with primary antibodies (1:1000) overnight at RT. After washing, the peroxidase-conjugated secondary antibody (1:1000) was applied for 1 h at RT. After extensive washing, the blots were developed using an enhanced chemiluminescence kit (GE Healthcare). Sample loading control was performed through β-actin immunodetection. The values of iNOS protein were normalized to those of β-actin.

### Nitrite assay

The culture medium was collected and centrifuged at 12,000 rpm for 5 min. Next, 250 µL/well of medium were treated with 20 µl of 6.5 M HCl and 20 µl of 37.5 mM sulfanilic acid in a 96-well plate. After incubation for 10 min, 20 µL of 12.5 mM N-(1-naphthyl)-ethylendiamine was added. Optical density was read at 550 nm after 15 min. Nitrite values were expressed as µmol nitrite/mg cell protein.

### Analyses of purine substrates and metabolites

Adenosine and its metabolites in VSMC culture medium were separated by high-performance liquid chromatography (HPLC) and quantified by UV absorption at 254 nm as previously described [27]. Briefly, medium samples were neutralized to a pH of 4–5 and mixed with 50 µM theophylline as internal standard. After centrifugation, supernatants were filtered through a 0.22 µm syringe filter. HPLC separation was achieved with a Beckman System Gold Programmable Solvent Module 125 on a Beckman C18 Analytical Ultrasphere column coupled to a Beckman Detector 166, using a non-linear NaH_2_PO_4_/tetrabutylammonium bromide (73.5/6 mM, pH 5.8)-methanol (0–25 %) gradient over 38 min, at a flow rate of 1 ml/min. The concentration of adenosine and its metabolites were extrapolated from calibration curves constructed through multiple determinations of standard amounts by plotting the peak areas against the concentration of the sample. Linearity of the assay was assessed using three calibration curves analyzed on separate days. The limit of detection and the lower limit of quantification were above 0.1 and 0.5 μM, respectively.

### Vascular reactivity

Changes in vasomotor tone of aortic tissues in response to adenosine were recorded in organ chambers. The thoracic aorta was carefully removed, cleaned of fat and connective tissue, and cut into 5–6 mm rings. Rings were suspended in Krebs-Henselheit solution with the following composition (mM): NaCl 118, KCl 4.7, KH_2_PO_4_ 1.2, MgSO_4_ 1.1, CaCl_2_ 2.5, NaHCO_3_ 25 and glucose 5.5; pH 7.4, maintained at 37 °C and continuously aerated with 95 % O_2_, 5 % CO_2_ for isometric tension recording in organ chambers. In some rings, endothelium was mechanically removed. The rings were connected to isometric tension transducers (Fort 10, World Precision Instruments) coupled with a digital recording system (PowerLab 8SP, ADInstruments). The completeness of endothelial denudation was confirmed by the absence of relaxation to the endothelial-dependent agonist acetylcholine (1 µM). After a 90-min equilibration period under a resting tension of 2 g, rings were constricted with 0.1 µM norepinephrine, and vasorelaxant responses to adenosine were determined in endothelium-denuded rings. When indicated, rings were previously incubated for 60 min either with vehicle (control) or the NO synthase inhibitor N^G^-monomethyl-l-arginine (L-NMA; 10 µM).

### Statistical analysis

Data are expressed as mean ± SEM, or as percentage, where appropriate. Comparison between 2 or more groups was performed using the Student’s *t* test and ANOVA, respectively. Linear correlations were checked using the Pearson’s r coefficient. Statistical analysis was accepted at *P* < 0.05 and SPSS ver. 21.0 was used.

## Results

### Influence of endogenous adenosine on iNOS synthesis and activity in VSMCs from diabetic rats and normoglycemic controls

**Fig. 1:**
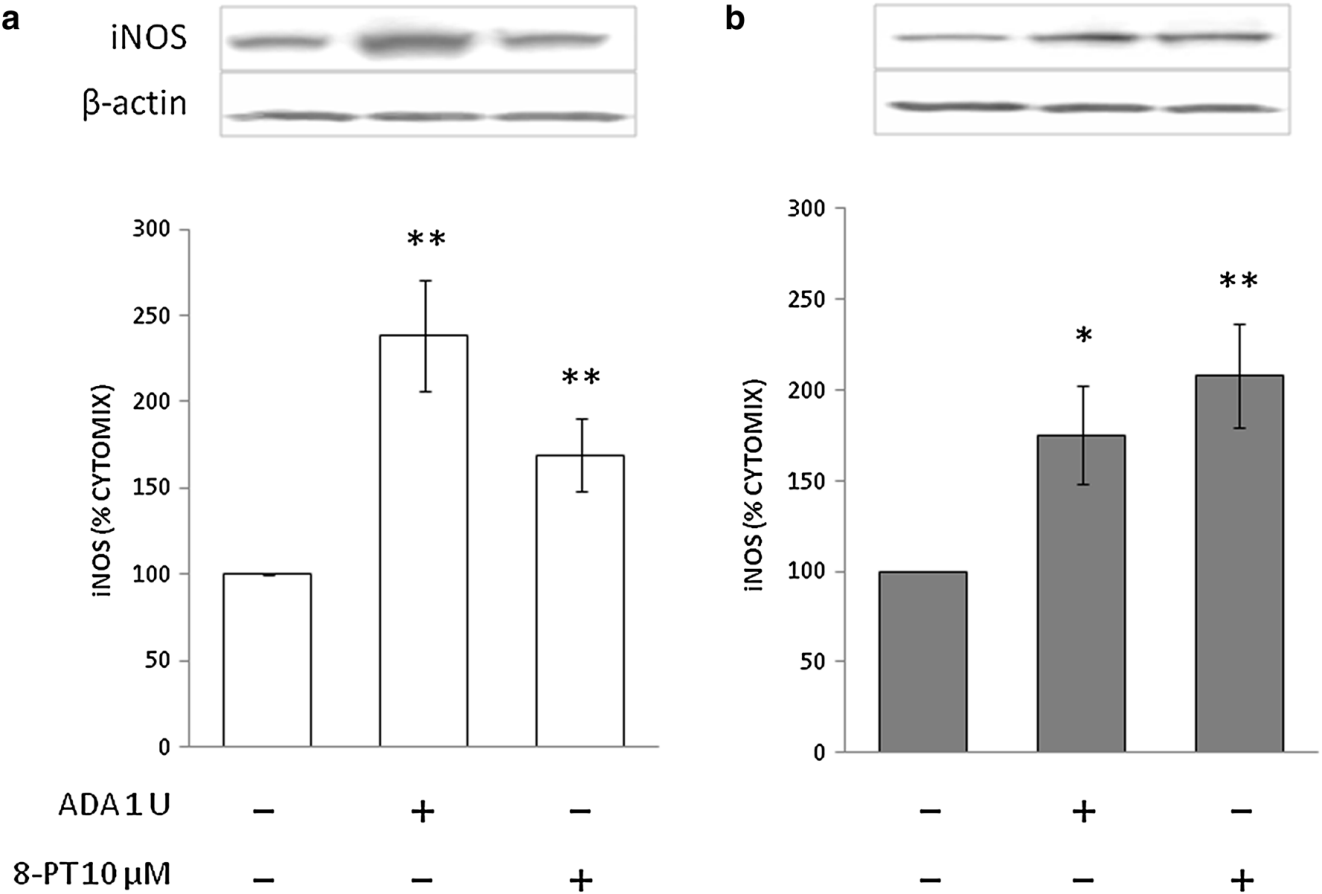
Immunoblots for iNOS in VSMCs from control (**a**) and STZ-diabetic rats (**b**) in the presence of endogenous adenosine modulators. VSMCs were incubated with cytomix comprising 10 ng/mL interleukin (IL)-1β, 10 ng/mL interferon (IFN)-γ and 25 ng/mL tumor necrosis factor (TNF)-α plus 1 µg/mL LPS for 24 h. Representative blots are shown. Densitometric analysis of iNOS protein levels are shown as % cytomix. The data are presented as mean ± SEM (*n* = 8). *P < 0.05, **P < 0.01 (two-tailed *t*-test). ADA, adenosine deaminase; 8-PT, 8-phenyltheophylline

We previously reported that iNOS expression and release of NO metabolites in response to 24-h stimulation with LPS and cytokines are attenuated by about 30 % in cultured VSMCs from STZ-diabetic rats as compared to those from normoglycemic rats [15, 28]. In the absence of inflammatory stimuli, iNOS is undetectable in these cells [15, 28]. This pattern was confirmed in the present study. In fact, at the end of a 24-h incubation of control or diabetic VSMCs in the presence of LPS and cytokines, which reproduce a setting of vascular inflammation, iNOS became detectable by Western blot (Fig. 1). Treatment with ADA to remove endogenous adenosine from the incubation medium enhanced the iNOS response to LPS/cytokines in both control and diabetic VSMCs. This effect was mimicked by the nonselective adenosine receptor antagonist 8-phenyltheophylline (8-PT; Fig. 1a, b).

**Fig. 2:**
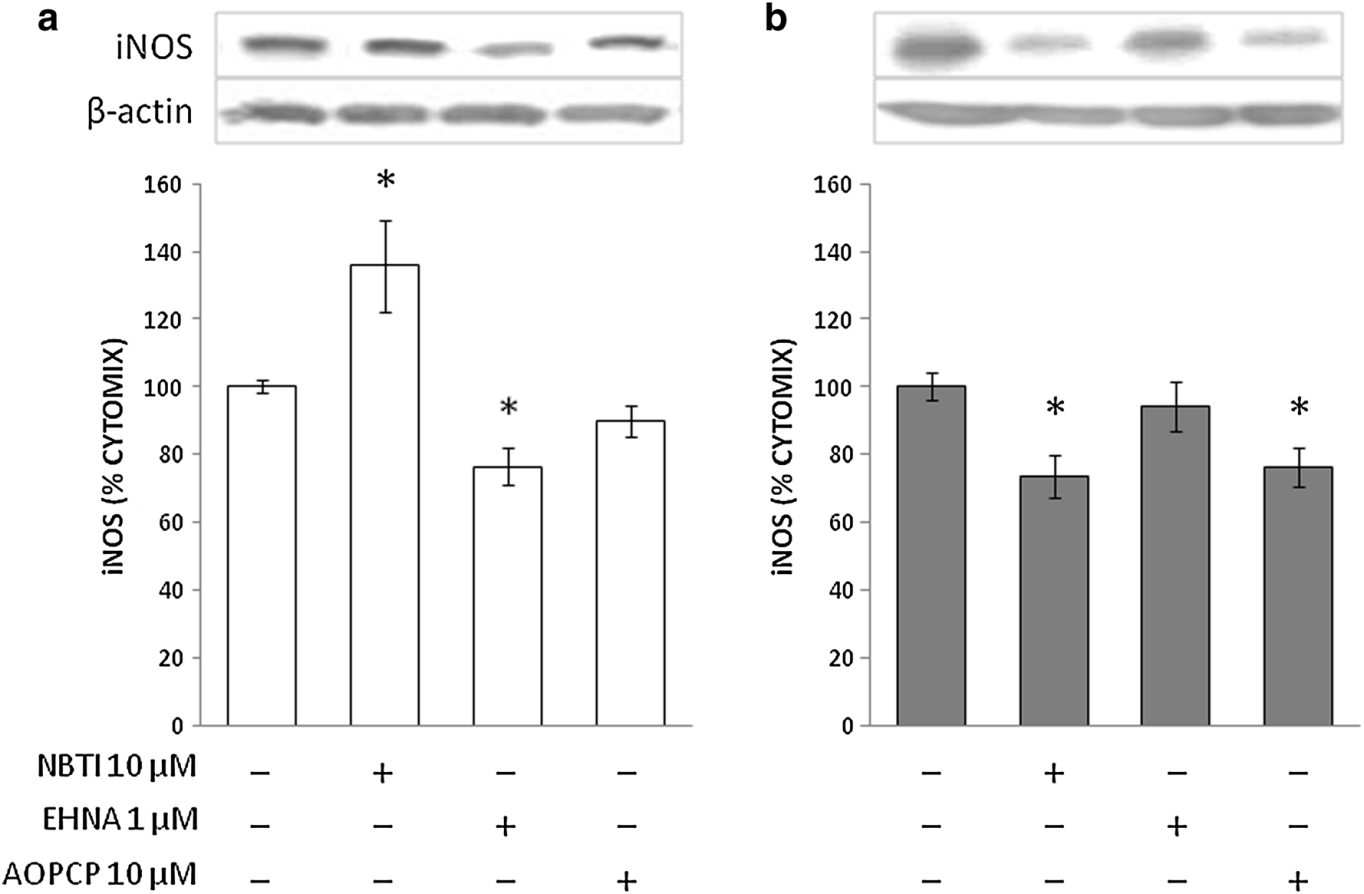
Immunoblots for iNOS in VSMCs from control (**a**) and STZ-diabetic rats (**b**) in the presence of adenosine turnover modulators. VSMCs were incubated as described in the legend to Fig. 1. Representative blots are shown. Densitometric analysis of iNOS protein levels are shown as  % cytomix. The data are presented as mean ± SEM (*n* = 8). *P < 0.05 (two-tailed *t*-test). NBTI, S-(4-Nitrobenzyl)-6-thioinosine; EHNA, *erythro*-9-(2-Hydroxy-3-nonyl)adenine; AOPCP, α,β-Methylene-ADP

In stimulated VSMCs from nondiabetic rats, the adenosine deaminase inhibitor *erythro*-9-(2-Hydroxy-3-nonyl)adenine (EHNA) reduced iNOS synthesis while the ENT inhibitor S-(4-Nitrobenzyl)-6-thioinosine (NBTI) enhanced it, and the CD73 inhibitor α,β-Methylene-ADP (AOPCP) was ineffective (Fig. 2a). In VSMCs from diabetic rats, under the same experimental conditions, EHNA did not cause any significant change in iNOS protein level. In contrast, AOPCP and NBTI partially prevented iNOS formation (Fig. 2b).

**Fig. 3:**
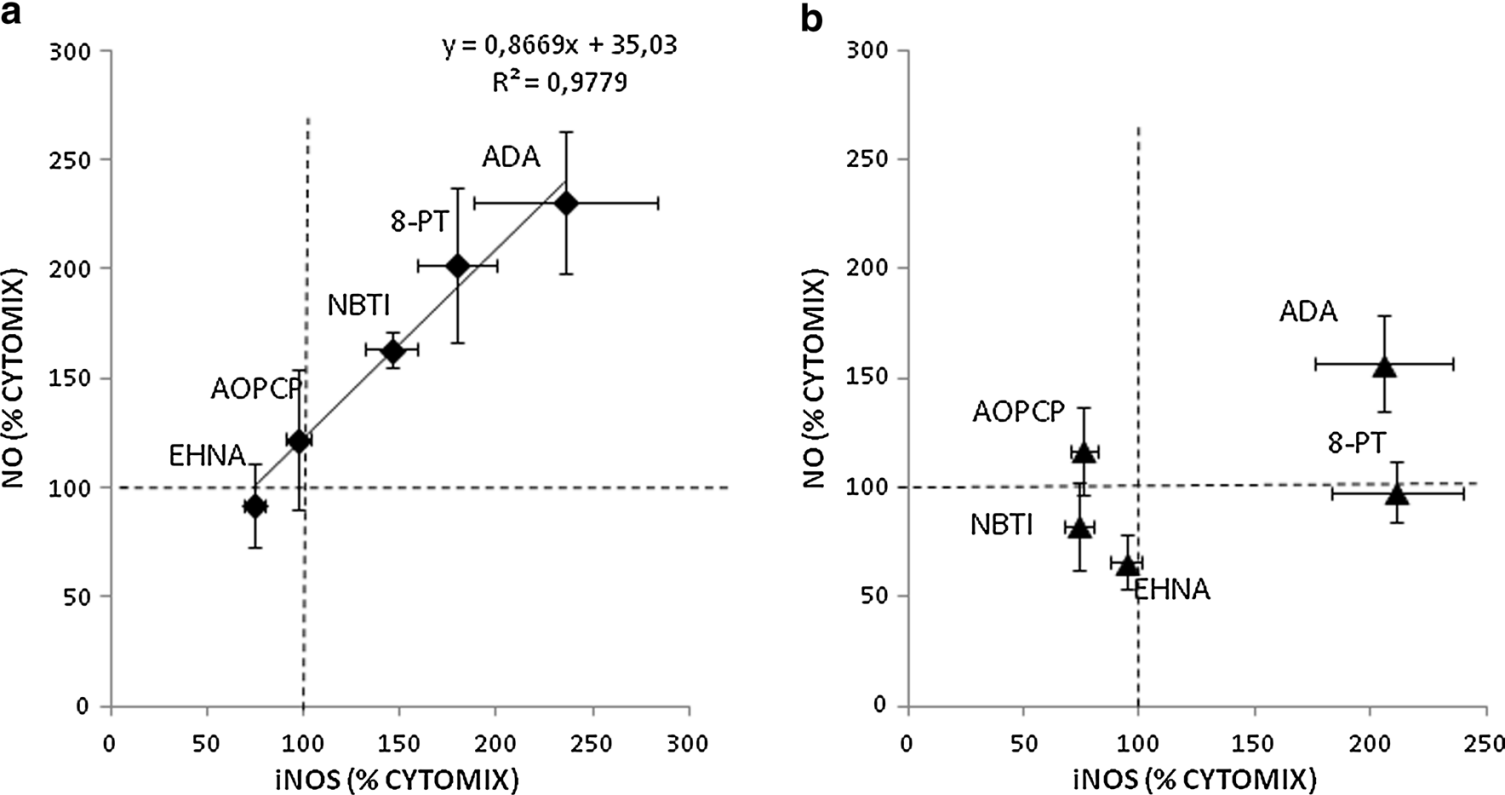
Correlations between iNOS levels and NO metabolite release into the culture medium of VSMCs from control (**a**) and STZ-diabetic rats (**b**). iNOS level data are from experiments shown in Figs. 1 and 2

In the above experiments, measurement of NO metabolites’ accumulation in the culture medium showed a direct and significant correlation between iNOS protein levels and enzyme activity in control VSMCs, which was disrupted in diabetic VSMCs (Fig. 3).

### Influence of exogenous adenosine on iNOS production

Under stimulation with LPS/cytokines, addition of adenosine did not alter iNOS levels in control VSMCs, while a significant 60 % increase was observed in diabetic VSMCs at the highest concentration tested (Table 1). In the presence of exogenous adenosine, iNOS protein levels were not affected by addition of EHNA or NBTI. Similarly, AOPCP did not alter the iNOS response to exogenous AMP (Table 1). Pretreatment of diabetic VSMCs with MRS1706 (0.1 μM, A_2B_ selective antagonist) significantly decreased adenosine-induced iNOS accumulation, in line with previous reports showing a prominent role for the A_2B_ receptor pathway [18, 19].

**Table 1:** Effects of exogenous adenosine/AMP on cytokine-induced iNOS synthesis in aortic smooth muscle cells

	Relative densitometric analysis of iNOS levels (arbitrary units)	
	Control (*n* = 7)	Diabetes (*n* = 8)
No addition	100 ± 8	100 ± 12
Adenosine 1 µM	95 ± 12	91 ± 7
Adenosine 10 µM	90 ± 12	114 ± 7
Adenosine 100 µM	97 ± 9	161 ± 14*
Adenosine 100 µM + EHNA 1 µM	112 ± 10	152 ± 14*
Adenosine 100 µM + NBTI 10 µM	93 ± 10	154 ± 22*
Adenosine 100 µM + MRS1706 1 µM^a^	90 ± 15	108 ± 11^**^
AMP 100 µM	108 ± 6	150 ± 12*
AMP 100 µM + AOPCP 200 µM	115 ± 9	176 ± 19*

Rat aortic smooth muscle cells from control and diabetic rats were incubated with 10 ng/mL interleukin (IL)-1β, 10 ng/mL interferon (IFN)-γ and 25 ng/mL tumor necrosis factor (TNF)-α plus 1 µg/mL LPS for 24 h. iNOS was detected by Western blot. Densitometric analysis of protein level is expressed as % of No addition

*EHNA erythro*-9-(2-Hydroxy-3-nonyl)adenine, *NBTI* S-(4-Nitrobenzyl)-6-thioinosine, *AOPCP* α,β-Methylene-ADP

* P < 0.01 vs. No addition, ** P < 0.05 vs. Adenosine 100 µM (two-tailed *t* test)

^a^ *n* = 3

**Fig. 4:**
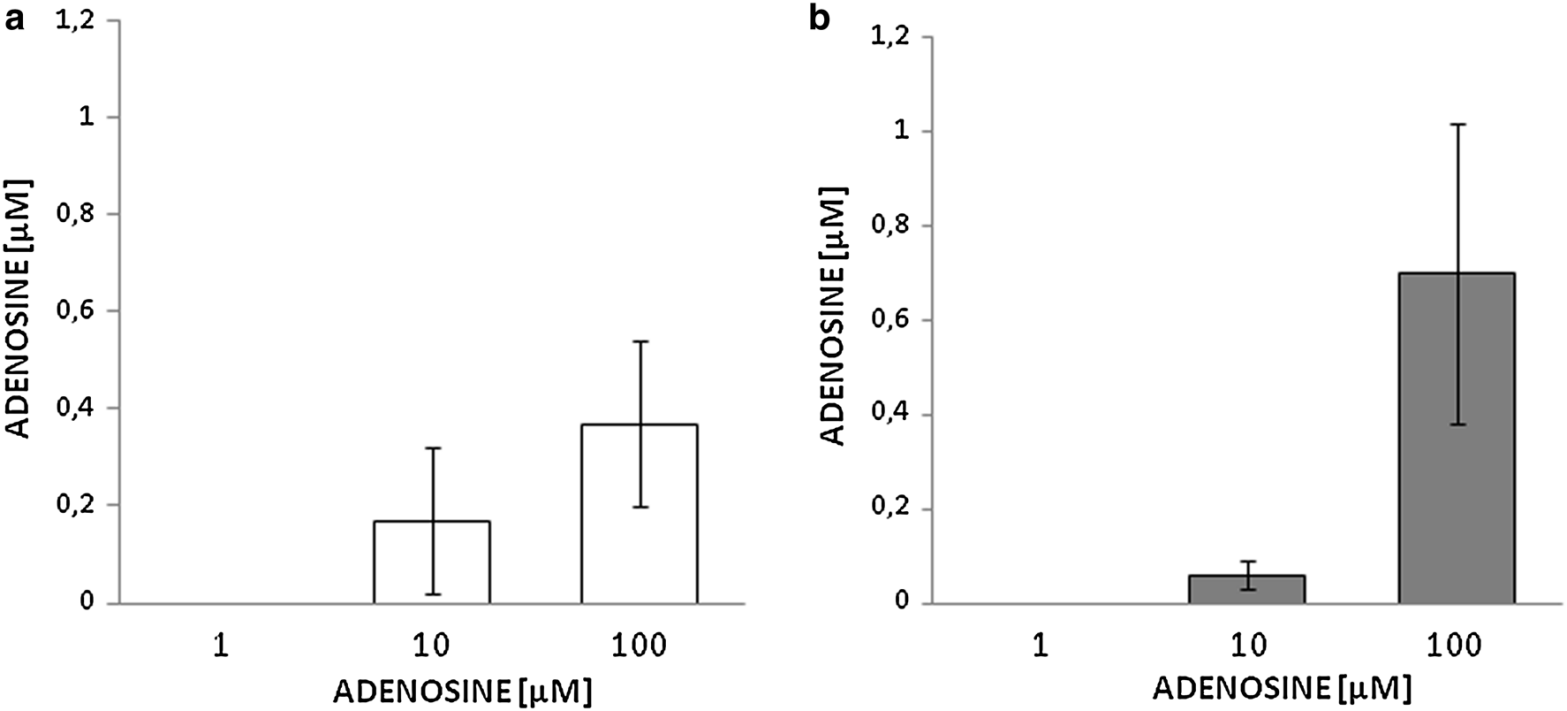
Residual adenosine concentration as measured by HPLC in the culture medium of VSMCs from control (**a**) and STZ-diabetic rats (**b**) following incubation with increasing adenosine concentrations (1–100 µM) for 24 h. The values represent the mean ± SEM (*n* = 10)

In these experiments the nucleoside measured in the medium after incubation was in the submicromolar range, often below the detection limit of our HPLC method (Fig. 4). When adenosine was measured immediately after its addition to the incubation medium (time zero, not shown), its recovery was close to 100 % (e.g. 99 ± 2 µM and 99 ± 1 µM in control and diabetic VSMCs, respectively, for 100 µM adenosine, *n* = 3).

**Fig. 5:**
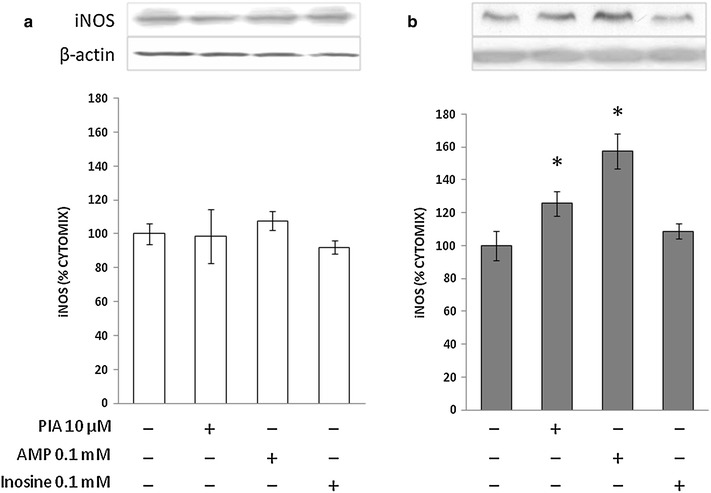
Immunoblots for iNOS in VSMCs from control (**a**) and STZ-diabetic rats (**b**) in the presence of exogenous purines. VSMCs were incubated as described in the legend to Fig. 1. Representative blots are shown. Densitometric analysis of iNOS protein levels are shown as % cytomix. The values represent the mean ± SEM (*n* = 5). *P < 0.05 (two-tailed *t*-test). PIA, N-(2-phenylisopropyl)-adenosine

Similar to 100 µM adenosine, the ADA-resistant adenosine derivative, phenylisopropyladenosine (PIA), as well as the direct precursor AMP increased iNOS protein level in diabetic VSMCs but were ineffective in those from control animals (Fig. 5). The deamination product of adenosine, inosine, did not alter iNOS levels in either cell type (Fig. 5).

### Turnover of exogenous adenosine

Cultured VSMCs release purine and pyrimidine nucleosides and their metabolites into external medium under basal conditions [5]. In the medium of VSMCs incubated for 24 h with or without LPS/cytokines, the only detectable metabolite was hypoxanthine: 14.2 ± 1.4 and 12.0 ± 3.6 µM, respectively, in control VSMCs; 11.7 ± 1.9 and 10.8 ± 2.1 µM, respectively, in diabetic VSMCs (*n* = 4).

**Fig. 6:**
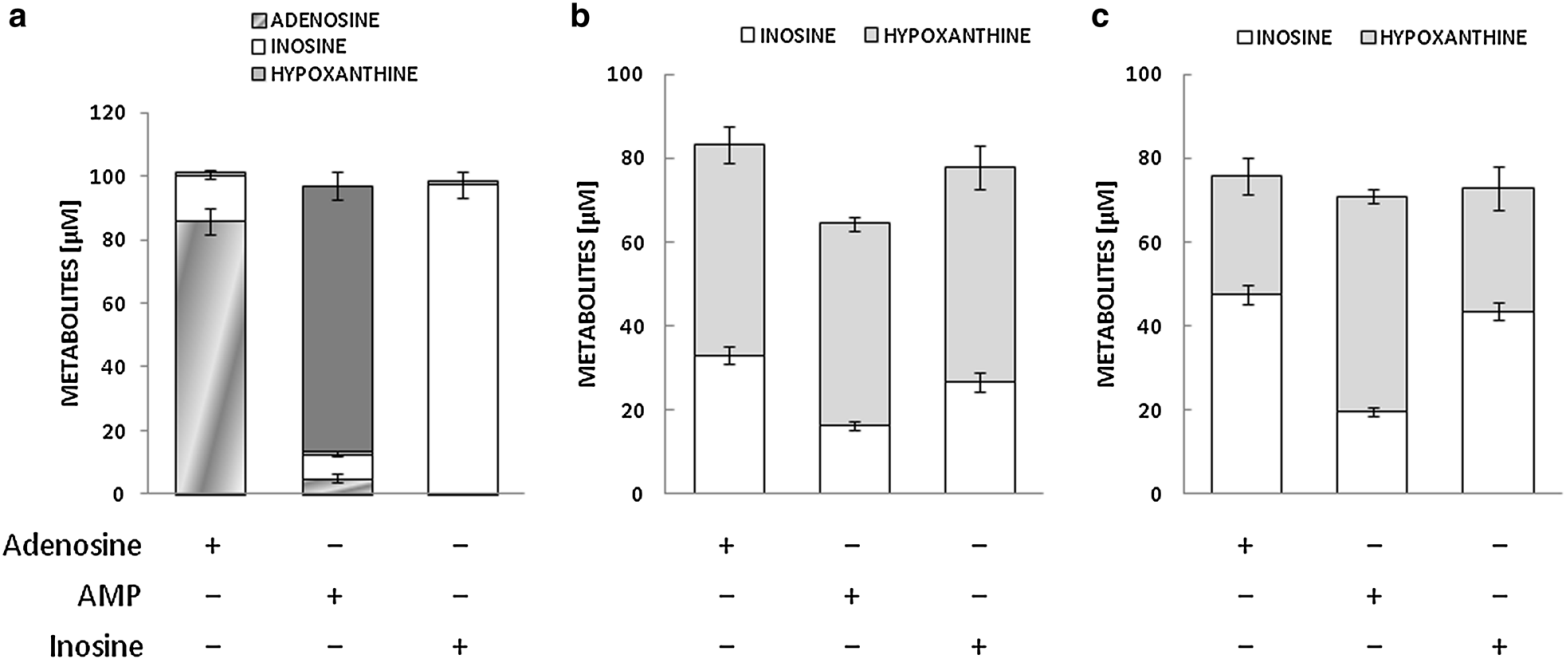
Residual concentration of adenosine and its metabolites as measured by HPLC following incubation with adenosine, AMP or inosine (all 100 µM) for 24 h in cell-free culture medium (**a**) or the culture medium of VSMCs from control (**b**) and STZ-diabetic rats (**c**). The values represent the mean ± SEM (*n* = 10)

As observed with adenosine (Fig. 4), AMP was efficiently cleared from the medium: both AMP and adenosine were undetectable after 24 h (Fig. 6). By contrast, exogenous inosine was only partially removed as its concentration accounted for more than 25 % of the nucleoside added at the beginning of the experiments in nondiabetic VSMCs, and this fraction increased to almost 45 % in diabetes (Fig. 6). Interestingly, for both cell types, these inosine concentrations were not significantly different from those recovered upon incubation with adenosine but were higher than those resulting from AMP degradation (Fig. 6). Adenosine, AMP and inosine were further converted into hypoxanthine, whose accumulation equalled 48–51 % of added substrates in control VSMCs. In diabetic VSMCs this fraction was comparable when adding AMP as a substrate, but decreased to less than 30 % when cells were incubated with adenosine or inosine (Fig. 6). The same incubations were performed also in the absence of cells, in order to detect spontaneous degradation and/or identify any bias related to possible ADA, 5′-nucleotidase and/or purine nucleoside phosphorylase (PNP) activity associated with FCS in the culture medium. Indeed, detectable amounts of inosine were also measured in samples incubated with AMP or adenosine. About half of exogenous AMP was converted to adenosine, while hypoxanthine concentrations did not differ from baseline levels (Fig. 6c). Treatment with pharmacological inhibitors did not enhance endogenous adenosine or AMP to detectable levels (data not shown).

**Fig. 7:**
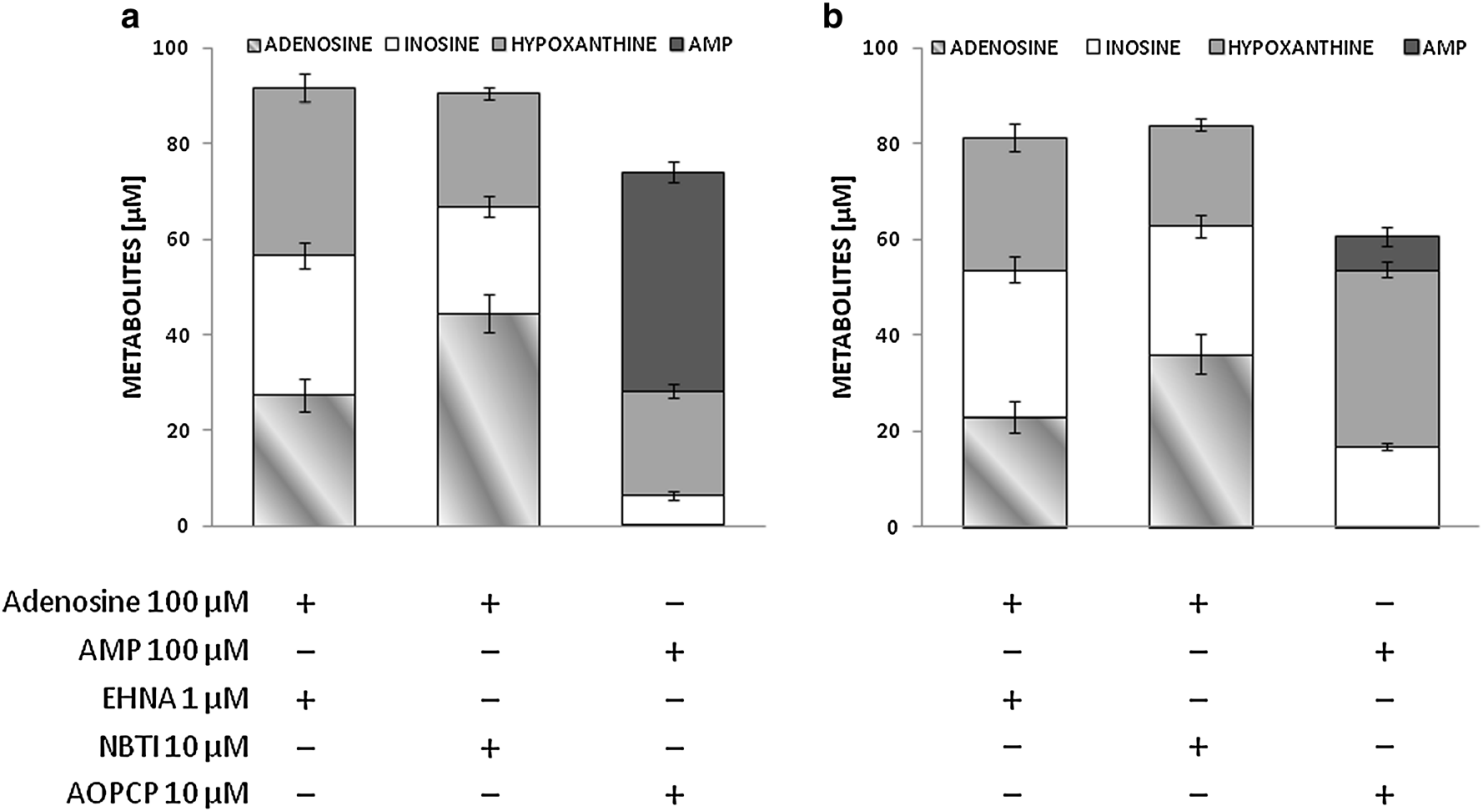
Residual concentration of adenosine, AMP and metabolites thereof as measured by HPLC following incubation with adenosine or AMP (both 100 µM) for 24 h in the culture medium of VSMCs from control (**a**) and STZ-diabetic rats (**b**). Inhibitors of adenosine/AMP turnover were added as indicated. The values represent the mean ± SEM (*n* = 6). NBTI, S-(4-Nitrobenzyl)-6-thioinosine; EHNA, *erythro*-9-(2-Hydroxy-3-nonyl)adenine; AOPCP, α,β-methylene-ADP

In the presence of exogenous adenosine, EHNA and, to a lesser extent, NBTI reduced the purine clearance, allowing its partial recovery in the medium, which was quantitatively similar for diabetic and control VSMCs (Fig. 7). By contrast, in those samples in which AMP was combined with AOPCP, the amount of recovered AMP accounted for half of the nucleotide added to control VSMCs, but for less than 10 % in experiments with diabetic VSMCs (Fig. 7), indicating that diabetes markedly reduced CD73 sensitivity to pharmacological inhibition by AOPCP.

### Adenosine-induced relaxation of endothelium-denuded aortic rings

**Fig. 8:**
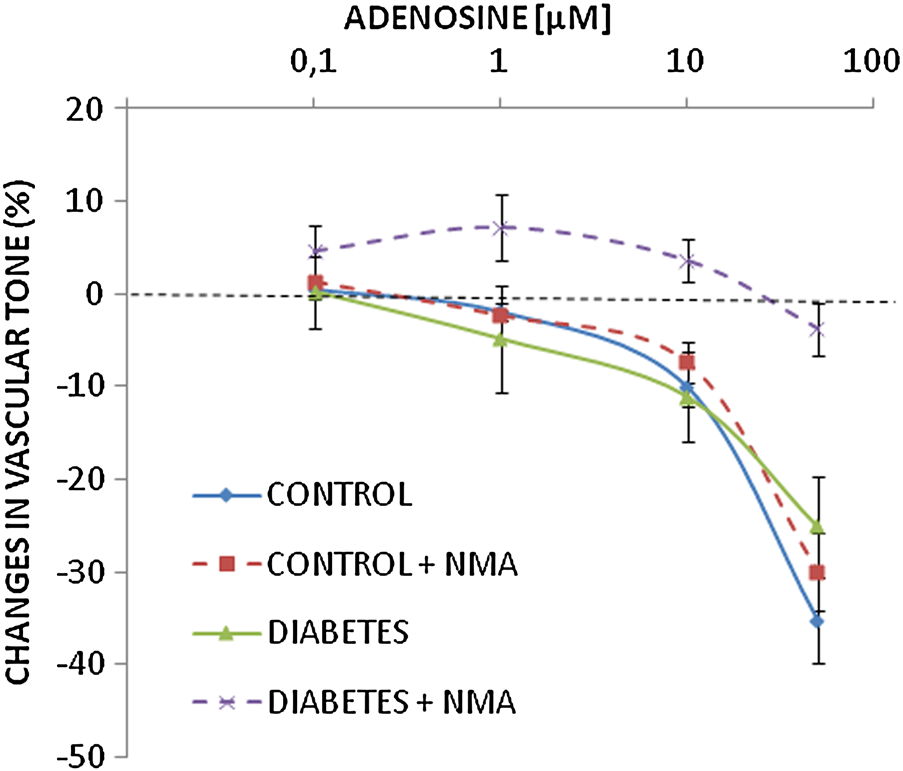
Concentration-response curves of endothelium-deprived aortic rings from control and STZ-diabetic rats precontracted with 0.1 µM noradrenaline and incubated with increasing concentrations of adenosine in the presence or absence of the NO synthase inhibitor L-NMA (10 µM). *n* = 5–7. Diabetes + NMA vs. diabetes, *P* < 0.001 (two-way ANOVA). Control vs. diabetes and control + NMA vs. control, *ns* not significant

In order to access a possible role of smooth muscle NOS in the vasorelaxant effect of adenosine, ex vivo experiments were performed on rings of endothelium-denuded rat aorta pre-contracted with norepinephrine. Under these conditions acetylcholine barely affected vascular tone (−10.5 ± 2.4, and −11.2 ± 4.9 %, change in norepinephrine-induced tension in preparations from control and diabetic rats, respectively; *NS*, *n* = 3), while adenosine significantly relaxed vascular tissues by up to 25.1 ± 5.4 and 35.3 ± 4.6 % in diabetic and control preparations, respectively (Fig. 8). Interestingly, pre-treatment with L-NMA abolished the vasorelaxant effect of adenosine in diabetic but not in nondiabetic aortic tissues (Fig. 8).

## Discussion

Diabetes affects the modulation of VSMC function by adenosine. For instance, primary cultures of aortic VSMCs obtained from rats with streptozotocin-induced diabetes mellitus have a greater susceptibility to the inhibitory effects of adenosine on cell proliferation [29]. Our results showed alterations in adenosine turnover and modulation of iNOS synthesis in the same cell model that can be relevant in the setting of diabetic vascular dysfunction.

### Regulation of iNOS functional expression by endogenous vs. exogenous adenosine

We found that endogenous adenosine impairs LPS/cytokine-induced iNOS formation in VSMCs, as shown by iNOS upregulation following treatment with ADA and with the nonspecific adenosine receptor antagonist 8-PT. Similarly, the ENT1 inhibitor NBTI enhanced iNOS accumulation in control VSMCs, in line with the observation that the adenosine uptake inhibitor dipyridamole up-regulates the IL-1β–induced NO production in a dose-dependent manner [30]. Under inflammatory conditions adenosine was released through ENTs in control VSMCs, whereas the transport direction appeared to be reversed in diabetic VSMCs. It has been speculated that increased ENT-1 activity as described in vitro in human SMCs exposed to hyperglycemia [31] or isolated from diabetic patients [32] may reduce adenosine availability to its receptors, thereby weakening the vascular functions of adenosine [10]. In diabetes, ENT-mediated transport is the main mechanism that cleared extracellular adenosine, while under control conditions ADA played a greater functional role. In the presence of the 5′-ectonucleotidase inhibitor AOPCP, extracellular AMP could achieve concentrations that mimicked the effects of adenosine on iNOS.

In contrast to previous reports [17–19], we were unable to detect increased iNOS functional expression by exogenous adenosine in VSMC from normoglycemic rats. Because either LPS or IL-1β alone were used to activate VSMCs in those studies, it is conceivable that the LPS/cytokine cocktail as used in the present study induced maximal iNOS activation via cyclic AMP [13] that could not be further enhanced by adenosine as described in the above-mentioned studies. However, we found that diabetes enhanced VSMC sensitivity to high concentrations of exogenous adenosine in terms of LPS/cytokine-induced iNOS production. To the best of our knowledge this finding is novel, and was likely determined by functional abnormality in Gi activity as described previously in VSMCs from diabetic rats [29] or activation of second messengers other than cAMP by high adenosine concentrations as suggested by others [17]. Because stimulated diabetic rat-derived VSMCs express less iNOS than control VSMC at earlier time points [15, 28] (Fig. 1), the increase in iNOS seen with 100 µM adenosine (Table 1) may be viewed as a short-term compensatory and beneficial effect. Whether exogenous adenosine affects the delayed response to long-term cytokine stimulation previously reported to occur in diabetic VSMCs [15] requires further investigation.

Unlike in control VSMCs, iNOS formation and NO release were found to be uncoupled in VSMCs from diabetic rats. Post-translational iNOS regulation may occur via several mechanisms including homodimerization by NOS-associated protein 1.10 kd (NAP110; [33]) or specific trafficking and activity regulation by multifunctional Ca^2+^/calmodulin-dependent protein kinase II (CaMKII; [34]). As the latter mechanism has been shown to be upregulated in VSMCs from diabetic rats [24], the observed uncoupling may be due to enhanced intracellular nitrotyrosine generation that is undetectable in the culture medium. This may represent a mechanism of cell/tissue injury involved in diabetic vascular dysfunction.

### Functional implications of perturbed NO levels in diabetes

Attenuated adenosine-mediated endothelium-dependent vasodilatation has been demonstrated in diabetic patients [35]. In agreement with previous studies [36], however, we found no abnormality in the relaxant response to adenosine of endothelium-denuded aortic rings due to diabetes. Our experiments in aortic rings not exposed to exogenous inflammatory stimuli (Fig. 8) suggest that smooth muscle NOS contributed significantly to adenosine-mediated vasorelaxation in vessels from diabetic but not nondiabetic rats, most likely due to increased iNOS expression in diabetic vessels [37, 38].

It should be pointed out that activation of NO synthase in diabetes is a multifactorial issue. For instance, overexpression of SIRT1 reduces diabetes-exacerbated myocardial ischemia-reperfusion injury and oxidative stress via activating eNOS in diabetic rats [39], and the vasculoprotective effect of insulin after arterial injury is mediated by an eNOS-dependent mechanism [40]. On the other hand, excess NO impairs glycemic control by diminishing insulin-stimulated muscle glucose uptake [41]. People with diabetes tend to have lower global levels of NO [42]. Therefore, beyond contributing to the homeostasis of the vasculature, NO is a key signaling molecule for insulin action and glucose disposal.

### Adenosine metabolism and turnover are altered in diabetic rat-derived VSMCs

With regard to major purine-inactivating pathways, ADA and CD73 were remarkably active in both control and diabetic VSMCs, with no residual AMP or adenosine found in the medium 24 h after supplementation. We also found a tendency to increased inosine generation following adenosine supplementation by diabetic VSMCs, possibly as a result of increased ADA activity, as described in diabetic patients [43] and in experimental models of hypertension [44]. Residual inosine concentrations following inosine supplementation to diabetic VSMCs were also higher than in control VSMCs. Thus, the enzyme PNP, which generates hypoxanthine from inosine, appeared to be the rate limiting step in adenosine catabolism, with somewhat lower activity in diabetic compared with control VSMCs. Yet supplementation of inosine, unlike that of adenosine, had no effect on iNOS synthesis in diabetic VSMCs. Overall, these findings point to alterations in the degradation pathways of exogenous purines in diabetes, which may have functional implications in VSMC function.

## Conclusions

To sum up, iNOS production induced by inflammatory stimuli was attenuated by endogenous adenosine in VSMCs, whereas addition of high concentrations of adenosine and its precursors/analogues enhanced iNOS production in diabetic compared with nondiabetic VSMCs. While it may be viewed as a compensatory and beneficial effect, the increase in iNOS mediated by exogenous adenosine was associated with abnormal purine turnover. Hence, NO-related alterations in adenosine metabolism and anti-inflammatory pathways may be involved in diabetic vascular dysfunction.

## Abbreviations

ADA: adenosine deaminase
AOPCP: α,β-Methylene-ADP
EHNA: *erythro*-9-(2-Hydroxy-3-nonyl)adenine
ENT: equilibrative nucleoside transporters
L-NMA: N^G^-monomethyl-l-arginine
LPS: lipopolysaccharide
NBTI: S-(4-Nitrobenzyl)-6-thioinosine
PIA: phenylisopropyladenosine
PNP: purine nucleoside phosphorylase
STZ: streptozotocin
VSMC: vascular smooth muscle cells
